# Commentary: Understanding intentions from actions: Direct perception, inference, and the roles of mirror and mentalizing systems

**DOI:** 10.3389/fnbeh.2016.00013

**Published:** 2016-02-11

**Authors:** Emmanuele Tidoni, Matteo Candidi

**Affiliations:** ^1^Istituto di Ricovero e Cura a Carattere Scientifico Fondazione Santa LuciaRome, Italy; ^2^Department of Psychology, Sapienza University of RomeRome, Italy

**Keywords:** action observation, action perception, intention understanding, mentalizing, transcranial magnetic stimulation, state-dependent TMS

Action perception, i.e., our ability to discriminate what action is performed and how, may underpin intention understanding (why an actor performed the observed action). Clearly, contextual cues do play a role in intention understanding but a crucial question is whether we are able to infer intentions by merely looking at actions (see Csibra and Gergely, 2007) and whether this function needs the activity of our motor system. In this commentary, we contend that (i) action discrimination is fundamental for intention understanding, (ii) this function needs the activity of the motor node of the AON, and (iii) further studies applying a causative approach to investigate the neural underpinnings of action discrimination and intention understanding are needed. Imagine observing a mime actor. His intentions (beside that of entertaining us) are realized through pantomimed actions and we need to discriminate how he grasps and manipulates imagined objects in order to understand his intentions.

In a recent article Catmur (2015) raised important questions about the role of the action observation network (AON) in the human ability to read others' mind. The author argued that the AON may support inferential processing providing sensorimotor information to mentalizing brain areas in order to make correct inferences about other's intentions, but that activity in the AON cannot fully account for our ability to understand others' intention. Catmur proposes a set of conditions that action observation neural responses should meet in order to determine the causal contribution of the AON in intention understanding. Namely: (1) the sight of an action must activate only one, matching, motor program in the observer; (2) this motor program must have a one-to-one mapping with the observer's own intention; (3) this mapping from motor program to intention must be the same in the observer as in the actor; and (4) upon activation of the motor program, the associated intention must be automatically activated, without the involvement of any higher-level inferential processes.

The first point relates to the functional connections between the visual and the motor system (Cattaneo et al., 2010; D'Ausilio et al., 2015), i.e., to what extent is our visual system able to discriminate between slightly different movements and whether this sensitivity is reflected in the activations of different patterns in the premotor counterpart of the AON (e.g., strictly or broadly congruent mirror neurons). In this respect it is important to note that causal studies have shown that interference/lesion with premotor regions impairs posture discrimination (Urgesi et al., 2007; Candidi et al., 2008; Moro et al., 2008).

The latter three points regard the functional relation between motor programs, inferential processes and the neural underpinnings of intention understanding. First we note that we are never able to produce exactly the same movement although we “select” the same motor program to realize one intention: i.e., there is no one-to-one mapping of intentions into motor programs. The third condition, we believe, should better leave some space for the natural variability in the ability to understand others' intentions by looking at their behavior: an intention may be implemented with different motor programs and two (or more) observers with different visuo-motor sensitivities may infer different intentions. The fourth point relates to the neural underpinnings of the processes. Whether action perception and intention understanding are carried out by the AON and Mentalizing areas separately is difficult to proof and the most reasonable answer is that (at least) the two systems work together. For example, contextual cues may help us generating an hypothesis about another person's intention (possibly requiring the activation of the Mentalizing network). However the accuracy of our hypothesis might be tested through the activity of the AON making us able to discriminate the observed actions.

The strongest evidence (although not to be considered conclusive, see Mahon and Caramazza, 2008) of a causal role of the AON for action discrimination comes from brain damaged patients (Urgesi et al., 2014) and brain stimulation interferential (e.g., TMS) studies (Avenanti et al., 2013). Crucially, the need for adequate control stimuli and control tasks (Press and Cook, 2015), and the necessity to have control sites of stimulation is becoming more and more recognized in the field as the understanding of the neural effects of brain stimulation techniques increases (Miniussi and Ruzzoli, 2013; Bestmann et al., 2015; Parkin et al., 2015).

Here we describe the contribution of one study applying some of the crucial controls that need to be implemented in experiments aimed at clarifying the contribution of fronto-parietal regions in action perception and actor's intentions understanding: namely, control tasks and sites of stimulation. In a series of TMS studies participants were asked to detect an actor's intention by observing truthful and deceitful actions characterized by slightly different movement kinematics (Tidoni et al., 2013). Here intention understanding pertains to the ability to infer the internal state of the actor based on the observation of his movements' kinematics. MEPs recorded from the observer during the observation of truthful and deceptive intentions showed a high degree of muscle specificity and a dynamic mapping of the observed actions' kinematics (Experiment 1). Crucially, by adopting an inhibitory repetitive-TMS approach the authors tested the role of the inferior frontal gyrus (part of the AON, Experiment 2 and 3) and the temporo-parietal junctions (part of the mentalizing network, Experiment 2) in the left hemisphere during an intention detection task. The results showed a reduced ability to discriminate between the two intents only when repetitive-TMS was applied over IFG and not when it was applied over TPJ. Importantly, the fact that no difference in participants' ability to discriminate visual stimuli was found in a difficulty-matched control task (Experiment 2 and 3) allowed the authors to exclude that the results were driven by a general inability to decode action-related visuo-spatial features (Press and Cook, 2015).

In conclusion, we agree with Catmur's suggestion that more causal evidences about the role of the AON and mentalizing system are necessary and we believe that novel methods of brain stimulation and the use of adequate control elements (e.g., brain areas, tasks) in future studies may greatly expand our understanding of the functional roles of these networks in the processing of action kinematics, goals and intentions understanding.

## Author contributions

All authors listed, have made substantial, direct, and intellectual contribution to the work, and approved it for publication.

### Conflict of interest statement

The authors declare that the research was conducted in the absence of any commercial or financial relationships that could be construed as a potential conflict of interest.
